# Association of hysterectomy with nonalcoholic fatty liver disease among US women

**DOI:** 10.1186/s12944-024-02020-4

**Published:** 2024-01-31

**Authors:** Shuanghong Jin, Shaoxun Li, Peipei Fang, Chenwei Pan, Shanshan Huang

**Affiliations:** Department of Infectious Disease, The Second Affiliated Hospital and Yuying Children’s Hospital of Wenzhou Medical University, 109 Xueyuan West Road, Wenzhou, 325027 China

**Keywords:** Hysterectomy, Nonalcoholic fatty liver disease, Menopause, Women

## Abstract

**Background:**

A postmenopausal rise in the rates of nonalcoholic fatty liver disease (NAFLD) has been reported in women. This study thus sought to further probe the association of hysterectomy with NAFLD.

**Methods:**

The data utilized in this investigation were attained from the 2017-March 2020 cycle of the National Health and Nutrition Examination Survey (NHANES), reflecting a strategic utilization of comprehensive health and nutrition information in the US population, to conduct a cross-sectional examination of the relationship between self-reported hysterectomy and NAFLD. Subjects included in this study were women aged 20 years or older. The multivariable logistic regression methodologies were utilized to determine the pertinent odds ratios (ORs) and their associated 95% confidence intervals (CIs).

**Results:**

Of the 2,868 subjects enrolled in this study (mean age: 51.3 years, 95%CI: 50.0-52.6 years), 22.1% (95%CI: 19.7–24.7%) reported having undergone a hysterectomy, while 31.1% (95%CI: 28.1–34.1%) exhibited elastographic evidence of NAFLD, and 3.8% (95%CI: 2.6–5.6%) exhibited clinically significant fibrosis (CSF). Relative to women with no history of hysterectomy, those that had undergone hysterectomy exhibited a higher odd of NAFLD (OR:1.66, 95%CI: 1.24–2.21) in a multivariable model fully adjusted for age, ethnicity, body mass index, female hormone use, oophorectomy, diabetes, hyperlipidemia, and smoking status. Subgroup analyses revealed a stronger association among women who were not obese (OR:2.23, 95%CI:1.61–3.11), women who were not affected by diabetes (OR:1.76, 95%CI: 1.25–2.46), and without hyperlipidemia (OR: 1.87, 95%CI: 1.10–3.16). No significant association of hysterectomy with NAFLD encompassing CSF was identified.

**Conclusions:**

The results of the present nationally representative analysis suggested an association between hysterectomy and increased NAFLD prevalence among US women. Knowledge of this relationship may better aid clinical efforts to screen for and manage NAFLD.

## Introduction

Nonalcoholic fatty liver disease (NAFLD) has become a prominent contributor to chronic liver disease worldwide, fueled by the escalating prevalence of type 2 diabetes and obesity in the general population [1]. NAFLD affects an estimated 30.05% of the global populace as well as 31.2% of North Americans [2]. NAFLD is associated with a risk of progressing to end-stage liver disease and hepatocellular carcinoma (HCC), particularly among patients affected by nonalcoholic steatohepatitis (NASH) [3]. NAFLD patients also generally face a wide range of metabolic comorbidities that place further strain on the healthcare system, making this condition a major public health challenge [4].

Epidemiologic evidence available at present indicates that NAFLD is more common among males relative to similarly aged women prior to menopause, whereas the incidence of this condition tends to rise among postmenopausal women [5, 6]. Both premature menopause and prolonged estrogen insufficiency have been linked to greater odds of hepatic fibrosis among postmenopausal women affected by NAFLD [7], and there are several reports suggesting that the risk of NAFLD increases among women who have undergone surgically induced menopause. A study from a UK database published in 2019 found that women with oophorectomy prior to age 50 had 37% elevated NAFLD risk [8]. In another research, women with endometrial cancer who had undergone oophorectomy similarly presented with a greater risk of NAFLD incidence (HR, 1.70) [9].

A majority of women who undergo surgical oophorectomy also have their uteruses removed. Indeed, hysterectomy is now believed to be the most common form of non-obstetrical surgery among US women with rates of up to 21.1%, leading to a risk of premature menopause in many of these treated women [10, 11]. Cumulating evidence suggests that hysterectomy with or without oophorectomy has adverse health consequences, such as increased risk of cardiovascular disease, hyperlipidemia, large artery stiffening, and stroke [12–16].

To date, no published studies have systematically explored the link between a history of hysterectomy and NAFLD among the general public. As such, this cross-sectional study sought to examine the potential association between hysterectomy and NAFLD among women based on data derived from the National Health and Nutrition Examination Survey (NHANES).

## Materials and methods

### Study design and participants

The 2017-March 2020 cycle of the cross-sectional NHANES survey performed by the US National Center for Health Statistics (NCHS) was used to conduct the present analysis. This survey employs a complex, multistage, probability-based approach to ensure the enrollment of a nationally representative sample of non-institutionalized civilians throughout the United States across all age groups. NHANES interviews are used to collect information regarding participant demographic, socioeconomic, and health-related characteristics [17]. Participants also undergo examinations that include both laboratory tests and medical measurements. Detailed information about the 2017-March 2020 data has been released publicly [18]. The implementation of all NHANES procedures received explicit authorization from the NCHS research ethics review board, and each participant, upon their survey admission, furnished written informed consent in adherence to the established protocol.

**Fig. 1:**
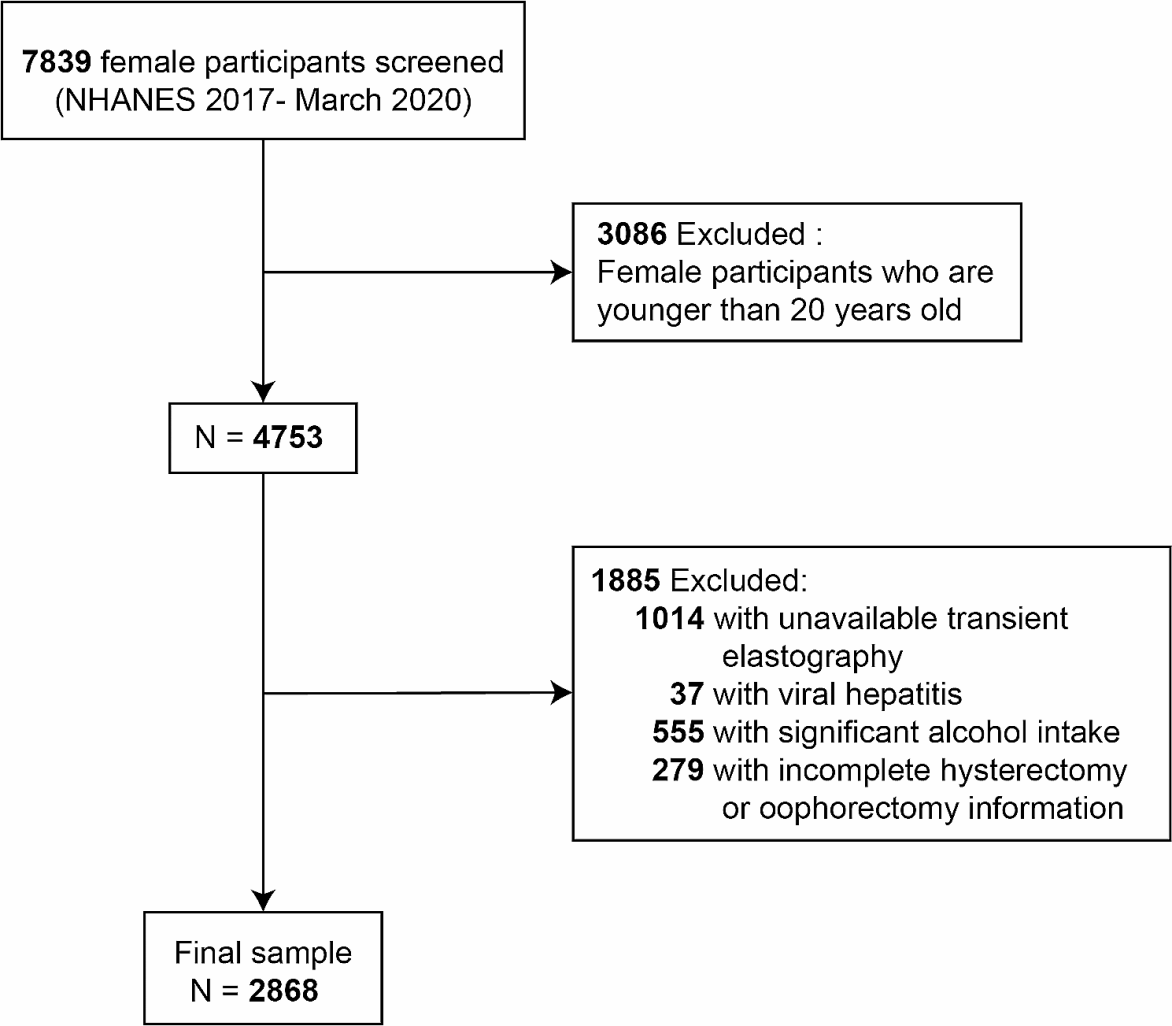
Flow chart for the selection and enrollment of study participants Abbreviations: NHANES, National Health and Nutrition Examination Survey

There were 7,839 total female subjects that participated in the NHANES 2017-March 2020 cycle. Of these individuals, those aged 20 years or older were selected for this study (*n* = 4753). Excluding those women without eligible vibration-controlled transient elastography (VCTE) results (*n* = 1,014), as well as those women who had a positive result for viral hepatitis B or hepatitis C infection (*n* = 37), women with a history of significant alcohol intake (average of 2 + standard drinks/day) (*n* = 555), and women for whom no information regarding hysterectomy or bilateral oophorectomy was available (*n* = 279), a final study sample of 2,868 subjects was obtained (Fig. 1).

### Reproductive factors

Each participant’s hysterectomy status was determined by her response to the inquiry: “Have you had a hysterectomy that is, surgery to remove your uterus or womb?”, with subjects that responded affirmatively being classified as having undergone a hysterectomy procedure. Other reproductive factors, including self-reported bilateral oophorectomy and female hormone use, were obtained from the questionnaire section (Reproductive Health) in the NHANES as part of the mobile examination center (MEC) interview.

### Transient elastography

Liver steatosis and fibrosis in NHANES participants were examined in the MEC through liver ultrasound transient elastography analyses conducted with a FibroScan model 502 V2 Touch instrument and a medium or extra-large probe based on the probe selection tool, as detailed previously [19]. Hepatic steatosis was recorded based on the controlled attenuation parameter (CAP), and liver stiffness measurement (LSM) was used to measure hepatic fibrosis. For the present study, a CAP ≥ 285 dB/m is considered consistent with liver steatosis, whereas an LSM ≥ 8.6 kPa is indicative of clinically significant fibrosis (CSF, fibrosis stage ≥ 2), which have been evaluated by Siddiqui et al. for its diagnostic performance to detect liver steatosis and fibrosis [20].

### Laboratory evaluations and other covariates

Detailed descriptions of the procedures utilized for laboratory evaluations have already been provided before [21]. Platelet count (PLT), albumin, alanine aminotransferase (ALT), aspartate transferase (AST), total bilirubin (TB), lipid profiles, hemoglobin A1c (HbA1c), and high-sensitivity C-reactive protein were included for analyses. Health technicians recorded the height, weight, and waist circumference (WC) of participants. Weight divided by the square of the height was used for the calculation of body mass index (BMI, kg/m^2^), of which ≥ 30 kg/m^2^ was considered as obesity. Diabetes mellitus (DM) was identified by previous physician diagnosis, taking prescribed medicine for DM, or HbA1c level of ≥ 6.5% [22]. Hyperlipidemia was identified based on a total cholesterol level of ≥ 200 mg/dL, HDL-C < 50 mg/dL, LDL-C ≥ 130 mg/dL, triglyceride level of ≥ 150 mg/dL, or taking prescribed medicine to decrease cholesterol [23]. The stratification of smoking status involved the categorization of participants into distinct groups: those who had never smoked (with an intake of fewer than 100 lifetime cigarettes), former smokers (having consumed more than 100 lifetime cigarettes but currently abstaining from smoking), and current smokers (having ingested more than 100 lifetime cigarettes and still actively engaging in smoking behaviors).

### Statistical analysis

All analyses were conducted using the weighting strategy recommended by the NCHS, presenting the continuous and categorical variables as weighted means and percentages, respectively, with corresponding confidence intervals (CIs). Survey-weighted linear regression or Chi-square tests were used to compare differences in these means and percentages, respectively, based on the NAFLD and hysterectomy status of participants.

The relationships between hysterectomy and NAFLD or NAFLD with CSF were assessed through multivariable logistic regression analyses adjusted for possible confounding factors including age, race/ethnicity, bilateral oophorectomy, female hormone use, BMI, diabetes mellitus, hyperlipidemia, and smoking status. Logistic regression model-based subgroup analyses were also performed based on participant age, female hormone use, obesity, diabetes, and hyperlipidemia status. R (v 4.2.0) and EmpowerStats (v 4.0) were used for all analyses, and it was attempted to regard a significance threshold when the two-tailed *P* fell below the threshold of 0.05.

## Results

### Participants’ characteristics

Initially, 7,839 female NHANES participants were screened for eligibility, with the ultimate involvement of 2,868 participants. These participants’ characteristics are accessible in Table 1. In total, 937 women (31.1%, 95%CI: 28.1–34.1%) exhibited elastographic evidence of NAFLD, including 122 participants (3.8%, 95%CI: 2.6–5.6%) who had clinically significant fibrosis. These participants had a mean age of 51.3 years (95% CI: 50.0-52.6 years) according to the weighted analysis. In addition, 663 had a self-reported history of hysterectomy, for an overall weighted prevalence of 22.1% (95% CI: 19.7–24.7%), while 12.3% (95% CI: 10.6–14.2%) reported having undergone bilateral oophorectomy.

**Table 1:** General characteristics of overall participants and according to the presence of NAFLD, NHANES 2017-March 2020

	Overall *n* = 2868	Non-NAFLD *n* = 1931	NAFLD *n* = 937	*P*-value
Age, years	51.3 (50.0, 52.6)	49.4 (48.1, 50.7)	55.4 (53.8, 57.0)	< 0.001
Race/ethnicity, %				0.401
NH-White	63.3 (57.7, 68.5)	63.4 (57.8, 68.7)	63.0 (55.4, 70.0)	
NH-Black	12.0 (9.1, 15.8)	12.4 (9.4, 16.2)	11.2 (8.0, 15.5)	
NH-Asian	6.2 (4.4, 8.7)	6.6 (4.8, 9.0)	5.3 (3.3, 8.4)	
Hispanic	14.7 (12.0, 17.9)	14.1 (11.4, 17.3)	16.0 (12.1, 20.9)	
other	3.8 (2.8, 5.0)	3.5 (2.3, 5.2)	4.5 (3.0, 6.6)	
BMI, kg/m^2^	29.7 (29.0, 30.3)	27.3 (26.7, 27.8)	35.0 (34.0, 35.9)	< 0.001
WC, cm	97.9 (96.4, 99.4)	92.1 (90.8, 93.4)	110.8 (108.7, 112.9)	< 0.001
Hysterectomy, %	22.1 (19.7, 24.7)	18.3 (16.3, 20.4)	30.4 (25.9, 35.4)	< 0.001
Bilateral oophorectomy, %	12.3 (10.6, 14.2)	10.3 (8.7, 12.2)	16.5 (12.7, 21.2)	0.002
Female hormone use, %	20.1 (17.9, 22.5)	18.3 (15.7, 21.3)	23.9 (20.4, 27.7)	0.009
Diabetes mellitus, %	13.7 (12.2, 15.4)	6.3 (5.2, 7.6)	30.2 (26.5, 34.2)	< 0.001
Hyperlipidemia, %	69.8 (66.5, 72.8)	63.5 (59.7, 67.1)	83.6 (80.1, 86.6)	< 0.001
Smoking status, %				0.784
Never	67.2 (64.6, 69.6)	67.5 (66.3, 69.7)	66.3 (60.7, 71.5)	
Former	21.5 (19.1, 24.1)	21.6 (19.7, 23.6)	21.4 (16.0, 27.9)	
Current	11.3 (9.5, 13.5)	10.9 (9.2, 12.8)	12.3 (8.6, 17.4)	
CAP, dB/m	254.8 (251.1, 258.4)	223.0 (220.4, 225.6)	325.4 (322.8, 328.0)	< 0.001
LSM, kPa	5.3 (5.1, 5.5)	4.7 (4.6, 4.9)	6.5 (6.0, 6.9)	< 0.001
Laboratory features				
ALT, IU/L	18.2 (17.6, 18.7)	16.5 (15.5, 17.5)	21.9 (20.9, 22.9)	< 0.001
AST, IU/L	19.5 (19.0, 19.9)	18.9 (18.3, 19.5)	20.6 (19.9, 21.3)	0.002
Total bilirubin, mg/dL	0.40 (0.39, 0.41)	0.41 (0.39, 0.42)	0.38 (0.37, 0.40)	0.061
Albumin, g/dl	4.03 (4.01, 4.05)	4.06 (4.03, 4.08)	3.98 (3.95, 4.01)	0.002
Total cholesterol, mg/dl	193.3 (190.4, 196.2)	192.3 (189.2, 195.5)	195.5 (191.3, 199.6)	0.146
HDL-cholesterol, mg/dl	59.3 (58.3, 60.3)	62.3 (61.2, 63.5)	52.6 (51.4, 53.9)	< 0.001
Triglyceride, mg/dl	124.9 (120.1, 129.3)	107.7 (103.4, 111.3)	162.8 (156.1, 169.5)	< 0.001
HbA1c, %	5.67 (5.64, 5.71)	5.48 (5.46, 5.51)	6.09 (6.01, 6.18)	< 0.001
Platelet count, 10^9^/L	258 (253, 264)	254 (249, 259)	268 (261, 276)	< 0.001
HS-CRP, mg/L	4.17 (3.68, 4.66)	3.41 (2.86, 3.97)	5.83 (5.19, 6.47)	< 0.001

Data are weighted means (95% CI) for continuous variables and weighted proportions (95% CI) for categorical variables. Differences by groups were assessed through Survey-weighted linear regression and Chi-square test

Abbreviations: NAFLD, nonalcoholic fatty liver disease; NHANES, National Health and Nutrition Examination Survey; NH, non-Hispanic; BMI, body mass index; WC, waist circumference; CAP, controlled attenuation parameter; LSM, liver stiffness measure. ALT, alanine aminotransferase; AST, aspartate aminotransferase; HDL, high-density lipoprotein; HbA1c: Hemoglobin A1c; HS-CRP, high-sensitive C-reactive protein

Women with NAFLD in the 2017-March 2020 NHANES cycle were older, exhibited higher BMI values, and higher frequencies of hysterectomy, bilateral oophorectomy, female hormone use, DM, and hyperlipidemia relative to women without NAFLD (*P* < 0.05).

Table 2 outlines participants’ characteristics categorized based on their hysterectomy status. Notably, women who had undergone hysterectomies displayed a significantly advanced age and manifested higher odds of belonging to the non-Hispanic White demographic. They also presented with higher BMI and WC values, together with greater frequencies of diabetes and hyperlipidemia (*P* < 0.05). These individuals also exhibited higher CAP and LSM values, as well as an increase in NAFLD prevalence (42.8%, 95%CI: 37.6–48.2% vs. 27.7%, 95%CI: 25.0-30.7%, *P* < 0.001), although no corresponding rise in the frequency of NAFLD with CSF was observed (5.4%, 95%CI: 3.0-9.6% vs. 3.4%, 95%CI: 2.3–4.9%, *P* = 0.088). Female hormone use was also more frequently reported among women with a history of hysterectomy (45.9%, 95%CI: 40.2–51.7%).

**Table 2:** General characteristics of participants according to the history of hysterectomy

	Without hysterectomy *n* = 2205	With hysterectomy *n* = 663	*P*-value
Age, years	48.0 (46.6, 49.4)	62.8 (61.3, 64.3)	< 0.001
Race/ethnicity, %			< 0.001
NH-White	61.6 (56.1, 66.8)	69.3 (61.5, 76.3)	
NH-Black	12.2 (9.5, 15.6)	11.4 (7.4, 17.0)	
NH-Asian	7.1 (5.1, 9.8)	3.0 (1.7, 5.2)	
Hispanic	15.7 (12.8, 19.2)	11.0 (7.8, 15.3)	
Other	3.3 (2.5, 4.4)	5.3 (3.2, 8.8)	
BMI, kg/m^2^	29.4 (28.7, 30.1)	30.6 (29.8, 31.4)	0.008
WC, cm	96.9 (95.3, 98.5)	101.5 (99.9, 103.2)	< 0.001
Bilateral oophorectomy, %	0.4 (0.2, 1.1)	54.1 (49.5, 58.7)	< 0.001
Female hormone use, %	12.7 (10.5, 15.4)	45.9 (40.2, 51.7)	< 0.001
Diabetes mellitus, %	10.8 (9.3, 12.5)	24.0 (19.7, 28.9)	< 0.001
Hyperlipidemia, %	66.9 (62.8, 70.7)	80.0 (76.2, 83.4)	< 0.001
Smoking status, %			0.344
Never	68.2 (65.0, 71.3)	63.4 (57.6, 68.9)	
Former	21.0 (18.3, 24.0)	23.3 (18.8, 28.5)	
Current	10.8 (8.6, 13.5)	13.3 (9.5, 18.4)	
NAFLD, %	27.7 (25.0, 30.7)	42.8 (37.6, 48.2)	< 0.001
NAFLD with CSF, %	3.4 (2.3, 4.9)	5.4 (3.0, 9.6)	0.088
CAP, dB/m	249.9 (245.8, 254.0)	272.0 (266.5, 277.6)	< 0.001
LSM, kPa	5.2 (5.0, 5.4)	5.6 (5.3, 6.0)	0.020
Laboratory features			
ALT, IU/L	17.7 (17.1, 18.2)	20.0 (17.8, 22.3)	0.076
AST, IU/L	19.2 (18.6, 19.8)	20.5 (19.5, 21.6)	0.058
Total bilirubin, mg/dL	0.40 (0.39, 0.42)	0.39 (0.36, 0.41)	0.314
Albumin, g/dL	4.04 (4.01, 4.06)	4.01 (3.98, 4.04)	0.166
Total cholesterol, mg/dL	191.1 (188.4, 193.8)	201.2 (195.1, 207.3)	0.001
HDL-cholesterol, mg/dL	59.1 (58.2, 59.9)	60.0 (56.9, 63.2)	0.555
Triglyceride, mg/dL	119.4 (115.5, 123.2)	144.7 (134.4, 155.0)	< 0.001
HbA1c, %	5.61 (5.57, 5.65)	5.90 (5.78, 6.01)	< 0.001
Platelet count, 10^9^/L	259 (254, 265)	255 (247, 263)	0.309
HS-CRP, mg/L	4.07 (3.57, 4.58)	4.51 (3.76, 5.26)	0.237

Data are expressed as weighted means (95% CI) for continuous variables and as weighted proportions (95% CI) for categorical variables. Differences by groups were assessed through Survey-weighted linear regression and Chi-square test

Abbreviations: NH, non-Hispanic; BMI, body mass index; WC, waist circumference; NAFLD, nonalcoholic fatty liver disease; CSF, clinically significant fibrosis; CAP, controlled attenuation parameter; LSM, liver stiffness measure. ALT, alanine aminotransferase; AST, aspartate aminotransferase; HDL, high-density lipoprotein; HbA1c: Hemoglobin A1c; HS-CRP, high-sensitive C-reactive protein

### Association between hysterectomy and prevalence of NAFLD

The association of hysterectomy with NAFLD was figured out through sample-weighted multivariable logistic regression analyses, as elucidated in Table 3. Women with a history of hysterectomy displayed a notably heightened susceptibility to NAFLD, as evidenced by an OR of 1.95 (95%CI: 1.58–2.40) when contrasted with their counterparts who had not undergone this surgical intervention. Following adjustment for a range of demographic and reproductive risk factors, this association persisted with some attenuation (OR: 1.62, 95%CI: 1.22–2.14), and it remained similarly strong when using a model that was fully adjusted for these factors as well as a range of metabolic risk factors (OR: 1.66, 95%CI: 1.24–2.21). However, no significant relationship between hysterectomy and NAFLD with CSF was observed through univariable or multivariable analyses of this population.

**Table 3:** Association of hysterectomy with NAFLD among women in the NHANES 2017- March 2020

Model	NAFLD			NAFLD with CSF	
	OR (95% CI)	*P*-value		OR (95% CI)	*P*-value
Model 1^a^	1.95 (1.58, 2.40)	< 0.001		1.62 (0.92, 2.84)	0.104
Model 2^b^	1.62 (1.22, 2.14)	0.002		1.38 (0.44, 4.28)	0.579
Model 3^c^	1.66 (1.24, 2.21)	0.005		1.41 (0.36, 5.46)	0.628

^a^ Crude model

^b^ Adjusted for age, race and ethnicity, bilateral oophorectomy and female hormone use

^c^ Adjusted for age, race and ethnicity, bilateral oophorectomy, female hormone use, BMI, diabetes, hyperlipidemia, and smoking status

Abbreviations: NAFLD, nonalcoholic fatty liver disease; NHANES, National Health and Nutrition Examination Survey; CSF, clinically significant fibrosis; OR, odds ratio; CI, confidence interval; BMI, body mass index

### Subgroup analyses

**Fig. 2:**
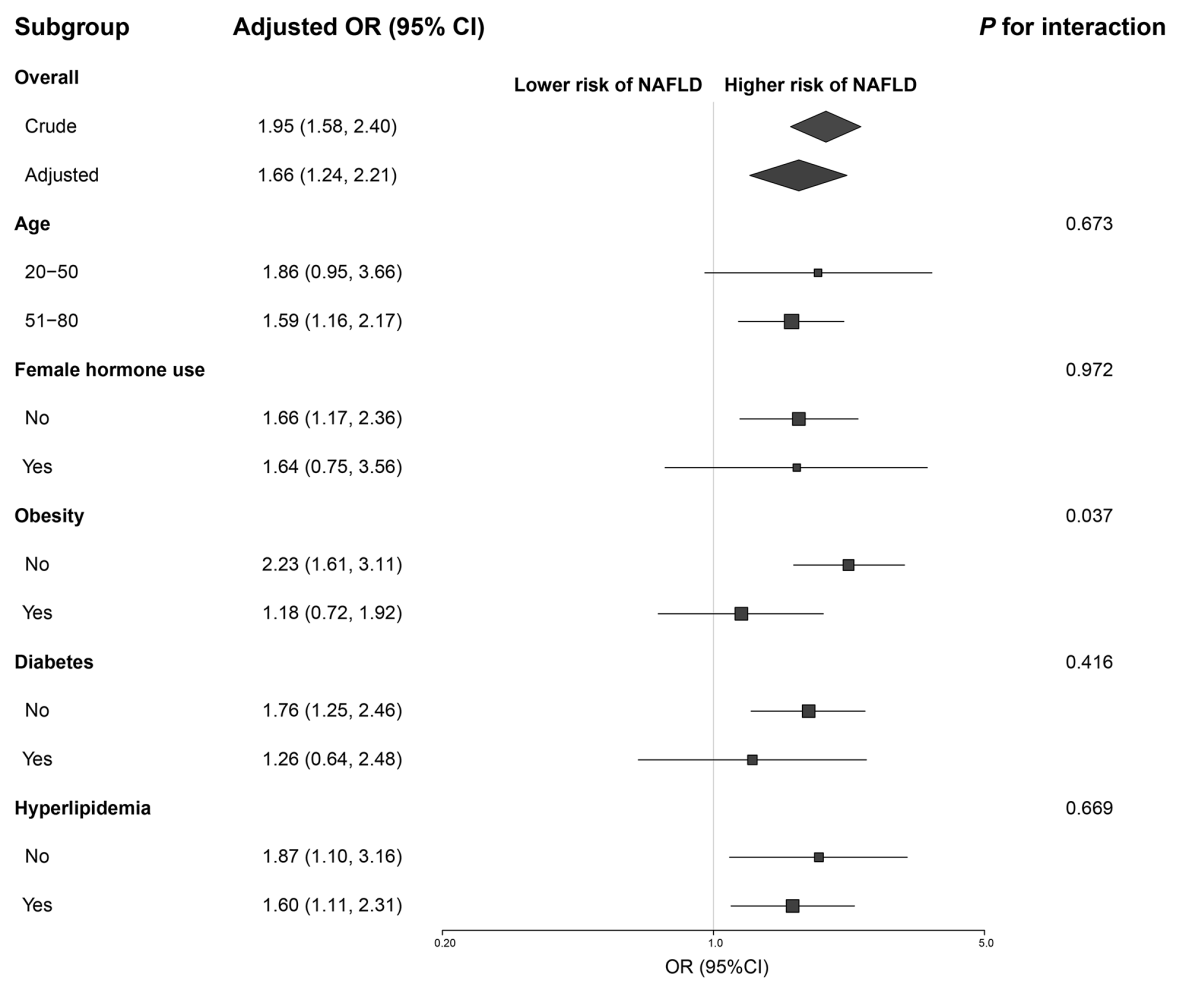
Association of hysterectomy with NAFLD in participant subgroups ORs and 95%CIs were derived from logistic regression models stratified for the indicated factors following adjustment for age, race and ethnicity, bilateral oophorectomy, female hormone use, BMI, diabetes, hyperlipidemia, and smoking status (with the exception of the factor used for stratification) Abbreviations: OR, odds ratio; CI: confidence interval; NAFLD: nonalcoholic fatty liver disease; BMI: body mass index

Further subgroup analyses are displayed in Fig. 2. A persistent association between hysterectomy and NAFLD was observed when stratifying subjects according to their hyperlipidemia status, with a slightly stronger association among non-hyperlipidemic individuals (OR: 1.87, 95%CI: 1.10–3.16). When stratified by age, female hormone use, obesity and diabetes, the association remained significant among women above 50 years (OR:1.59, 95%CI:1.16–2.17), women without hormone use (OR:1.66, 95% CI: 1.17–2.36), non-obese women (OR:2.23, 95%CI:1.61–3.11) and non-diabetic women (OR:1.76, 95%CI: 1.25–2.46). However, the association was not significant among women aged 50 years or younger, who received female hormones, with diabetes, or in the obesity subgroup. Obesity status also significantly influenced the effect linking hysterectomy and NAFLD, with a greater risk observed in women without obesity (*P* for interaction < 0.05).

## Discussion

This study stands as the pioneering population-based investigation uniquely dedicated to figuring out the intricate relationship between hysterectomy and the occurrence of NAFLD. Hysterectomy was found to be related to a higher prevalence of NAFLD among this nationally representative US population, with persistent association observed after adjustment for bilateral oophorectomy, female hormone use, and various metabolic risks. This association was also found to be stronger among women without obesity, those who did not have diabetes and hyperlipidemia.

This research performed a cross-sectional analysis utilizing data attained from the NHANES 2017-March 2020 cycle, wherein individuals from the broader general public were actively involved as participants. In prior studies evaluating the relationship between oophorectomy and NAFLD, the diagnosis of NAFLD was extracted from the medical records [8, 9, 24], which may underestimate the real prevalence of NAFLD. VCTE was herein used to objectively assess NAFLD status, as this technique has been validated to accurately detect fatty liver and fibrosis, even in the early asymptomatic stages of the disease. Given the potential of oophorectomy and female hormone therapy to confound the results of this study, these factors were adjusted for in multivariable analyses, yet the relationship between hysterectomy and NAFLD remained significant. These findings are in line with those published by Wang et al. [24], who noted a higher risk of NAFLD among women that reported a history of simple hysterectomy in a subgroup analysis of their study of menopausal women. Certain cohort studies have also detected a relationship between hysterectomy and a greater risk of liver cancer [25, 26]. Fibrosis grade has been linked to the occurrence of HCC [27]. In this setting, the association of hysterectomy with significant fibrosis was evaluated. Women with hysterectomy had higher values of LSM than their counterparts without, but no statistical association between hysterectomy and NAFLD with CSF was detected. However, the relatively limited number of women with significant fibrosis in this study population made it difficult to reach statistical significance owing to the wide confidence intervals.

Previous data have indicated the correlation of a history of hysterectomy with worse lipid profiles and higher rates of diabetes and obesity [13, 28]. Align with prior studies, individuals with hysterectomy in this population also displayed a higher BMI and elevated rates of diabetes and hyperlipidemia, all of which are risk factors for hepatic steatosis, potentially explaining the higher odds of NAFLD among these women. Following the adjustment for metabolic risk factors in multivariable analyses, the observed association of hysterectomy with NAFLD was still significant. In addition, further subgroup analyses indicated the association was stronger in women without obesity, diabetes, and hyperlipidemia. These findings also align with previous data derived from Clinical Practice Research Datalink [8], which highlighted the more noticeable risk of NAFLD among non-diabetic women who had undergone oophorectomy in the UK population. In light of these findings, it appears that the increased odds of NAFLD associated with hysterectomy are more common in women with fewer components of metabolic syndrome, emphasizing a need to screen for NAFLD in these subgroups of individuals typically regarded as being at low risk.

There are several possible explanations for the relationship of hysterectomy with NAFLD detected herein. As a sexually dimorphic organ, androgens and estrogens could interfere with many metabolic processes in the liver [29–31]. The procedure of hysterectomy has been reported to disrupt the ovarian blood flow and remove paracrine signals of the endometrium, with consequences for premature ovarian failure and lower estrogen production [32, 33]. Additionally, women experience a significant decline in estrogen and androgen production after bilateral oophorectomy. However, hysterectomy with ovarian preservation relatively reserves androgen production after surgery, which may lead to a shift toward androgen predominance [34, 35]. Exposure to androgens is reportedly associated with the incidence of hepatic steatosis and insulin resistance [36]. Following the hormone transition of hysterectomy, adverse changes in lipid metabolism may promote hepatic accumulation of fat and cholesterol.

An additional possible explanation could be the iron overload due to hysterectomy. Postmenopausal women have increased iron stores, and have the potential to experience iron overload after cessation of menstrual blood loss [37, 38]. Accumulating evidence has indicated that increased iron stores and impaired iron homeostasis could contribute to the development of NAFLD by insulin resistance and overproduction of reactive oxygen species [39, 40]. However, the precise molecular mechanisms underlying hysterectomy and NAFLD are still required for further investigations.

### Strengths and limitations

This study encompasses notable strengths and limitations, necessitating careful acknowledgment and examination to comprehend its implications. As it enrolled a nationally representative participant sample and included transient elastography-based analytical results, it has the potential to offer more accurate insight into the true prevalence of NAFLD as compared to studies based on medical records. In addition, a wide range of confounding factors with the potential to impact NAFLD risk were taken into consideration in this study, including bilateral oophorectomy, female hormone use, and metabolic factors, yielding consistent results. However, this study involves specific limitations that should be pointed out. As a cross-sectional analysis, the potential causal and temporal association between hysterectomy and NAFLD could not be established herein. As previously found by Howard et al., women who undergo hysterectomy tend to have more obesity and diabetes at baseline in their study [41]. Further well-designed cohort studies will be vital to address this issue. Another limitation is the status of hysterectomy and/or oophorectomy was identified by self-reported data such that these results may be less accurate than analyses conducted based on medical records. Previous literature, however, has demonstrated that self-reported hysterectomy had high accuracy and validity when compared with medical record reviews or ultrasound results [42, 43]. Lastly, the NHANES database did not provide information regarding the age at hysterectomy, precluding any possible assessment of the impact of this parameter on the observed link between hysterectomy and NAFLD.

## Conclusions

In conclusion, the outcome of this nationally representative population-based investigation revealed that hysterectomy was associated with higher odds of NAFLD among US women, with this relationship persisting even following adjustment for relevant confounders. In subsets of the study population, this increased odds of NAFLD were more prominent in hysterectomized women with fewer metabolic abnormalities. These data expand prior findings of potential health risks following hysterectomy and strengthen the understanding of hormonal milieu perturbation in the development of NAFLD. Given that hysterectomy is a relatively prevalent procedure among US women, targeted NAFLD screening and interventional efforts should be provided to these individuals to help address the risk observed herein.

## Data Availability

The NHANES data are publicly available for researchers provided by NCHS of the Centers for Disease Control and Prevention (https://www.cdc.gov/nchs/nhanes).
